# Quantitative imaging assessment of blood-brain barrier permeability in humans

**DOI:** 10.1186/2045-8118-10-9

**Published:** 2013-02-07

**Authors:** Yoash Chassidim, Ronel Veksler, Svetlana Lublinsky, Gaby S Pell, Alon Friedman, Ilan Shelef

**Affiliations:** 1 Departments of Physiology and Cell Biology & Biomedical Engineering, Faculty of Health Sciences, Zlotowski Center for Neuroscience, Ben-Gurion University of the Negev, Beer-Sheva, Israel; 2 Department of Radiology, Soroka University Medical Center and Zlotowski Center for Neuroscience, Ben-Gurion University of the Negev, Beer-Sheva, Israel; 3 Department of Life Sciences, Ben-Gurion University of the Negev, Beer-Sheva, Israel; 4 Brainsway Ltd, Jerusalem, Israel

**Keywords:** Blood–brain barrier, Magnetic resonance imaging, Permeability, Brain insult

## Abstract

The blood–brain barrier (BBB) is a functional and structural barrier separating the intravascular and neuropil compartments of the brain. It characterizes the vascular bed and is essential for normal brain functions. Dysfunction in the BBB properties have been described in most common neurological disorders, such as stroke, traumatic injuries, intracerebral hemorrhage, tumors, epilepsy and neurodegenerative disorders. It is now obvious that the BBB plays an important role in normal brain activity, stressing the need for applicable imaging and assessment methods. Recent advancements in imaging techniques now make it possible to establish sensitive and quantitative methods for the assessment of BBB permeability. However, most of the existing techniques require complicated and demanding dynamic scanning protocols that are impractical and cannot be fulfilled in some cases. We review existing methods for the evaluation of BBB permeability, focusing on quantitative magnetic resonance-based approaches and discuss their drawbacks and limitations. In light of those limitations we propose two new approaches for BBB assessment with less demanding imaging sequences: the *“post-pre”* and the *“linear dynamic”* methods, both allow semi-quantitative permeability assessment and localization of dysfunctional BBB with simple/partial dynamic imaging protocols and easy-to-apply analysis algorithms. We present preliminary results and show an example which compares these new methods with the existing standard assessment method. We strongly believe that the establishment of such “easy to use” and reliable imaging methods is essential before BBB assessment can become a routine clinical tool. Large clinical trials are awaited to fully understand the significance of BBB permeability as a biomarker and target for treatment in neurological disorders.

## Review

The blood–brain barrier (BBB) is a tightly-regulated, structural and functional barrier that controls the extracellular neuronal environment within the brain and spinal cord by limiting the free passage of ions and large molecules into the CNS. BBB functions are often impaired in common neurological disorders, including stroke, traumatic injuries, intracerebral hemorrhage, tumors, epilepsy and neurodegenerative disorders. BBB dysfunction is often associated with increased vascular permeability to plasma constituents, including large proteins, and results in water influx and brain edema [1]. Recent animal experiments demonstrated that serum proteins may serve as direct signaling mechanism within the brain resulting in the activation of astrocytes and the brain immune system, with a consequent neuronal hyperexcitability and delayed neurodegeneration [2]. These studies have highlighted BBB dysfunction as a potential biomarker for neurological disorders, with the possibility for predicting complications and neurological outcome after an insult and for determining novel treatments. Recent advancements in imaging methods now make it possible to establish sensitive and quantitative methods for the assessment of BBB permeability, most of which are MRI based.

Methods based on magnetic resonance imaging (MRI) scans following intravenous injection of MR-visible contrast agents containing gadolinium are currently the gold standard and most commonly used non-invasive imaging technique for the assessment of BBB impairment in both clinical [3-5] and preclinical studies [6]. One of the main advantages of MRI is the ability to produce multi-parametric information, allowing high-resolution anatomical information, and assessment of cerebral blood flow (CBF) and BBB integrity using the same imaging modality [7]. BBB assessment and permeability quantification are carried out by either using semi-quantitative methods, where statistical differences between scans before and after tracer injection are calculated [8], or by using more quantitative approaches based on a dynamic contrast enhanced MRI (DCE-MRI) [9,10]. These approaches assess the kinetics of a contrast agent over time and space to detect regions with increased vessel permeability. However, most of the existing techniques require complicated and demanding dynamic scanning protocols, including tracer injection during the scan and long scanning sessions. These imaging protocols in addition to the complex and computationally-demanding assessment algorithms, make these dynamic methods impractical and in some cases even unfeasible, stressing the need for developing “lighter” and less demanding semi-quantitative methods. One limitation occurs particularly in cases where patients need to be scanned immediately following a treatment that cannot be performed inside the magnet, preventing tracer injection during the scanning as required in DCE-MRI. We describe and discuss two new methods with less demanding MRI protocols and easy-to-apply analysis algorithms, the *post-pre* and *linear dynamic* methods. In the post-pre method only two scans are statistically compared and analyzed, one scan before the tracer injection and the other after the tracer injection. Regions with statistically-significant positive changes are marked as potentially permeable and further processed. The linear dynamic method is a semi-dynamic approach, where scans are taken at several time points after the tracer injection and analyzed according to their linear slope. Both proposed methods overcome some of the limitations described while supplying semi-quantitative assessment comparable to the existing more complex method.

## Methods

We describe two practical approaches for BBB assessment using DCE-MRI with simpler imaging sequence requirements: (1) The “*post-pre comparison*” method, in which a statistical comparison is performed between pre- and post-contrast agent injections; and (2) The “*linear dynamic method*”, where the dynamics of the signal change in multiple scans after contrast agent injection are calculated, assuming a linear model. Additional background information on the dynamic DCE-MRI method can be found in the Appendix. The protocol for this study was approved by the Soroka University Medical Center Ethics Committee; a written informed consent was obtained from all participants. More details on the

(1) Post-pre comparison

The post-pre approach is based on a comparative analysis between contrast-enhanced MR scans before and after the intravenous contrast agent injection. Scans acquired after the injection show regions with enhanced contrast agent, which can be quantified by comparison with the corresponding pre-injection non-contrast scans. Here we present an advanced method for permeability assessment based on statistical analysis of the imaging.

T1-weighted sequences are taken before and 5 minutes after the injection of Gd-DTPA with dose normalized according to patient’s body weight (Gd-DTPA 0.5M, 0.1mmol/kg). The scans are mapped to a standard brain atlas to avoid artifacts due to head movements and to allow accurate pixel based comparison between scans and even between patients for group studies analysis. This can also be achieved by co-registration between the pre- and post-injection images. Regions outside the brain are excluded from the images and the brain region is segmented into three tissue classes: gray matter, white matter and cerebrospinal fluid (CSF). A brain tissue segment is defined by the combination of the gray and white matter regions.

Pre- and post- contrast images are compared in several steps. First, significantly different pixels are identified by performing a slice-wise unpaired t-test (slice-wise processing was chosen for images with non-isometric voxel size). To this goal, a neighborhood comparison is applied where for each pixel, the 3x3 neighborhood (nine pixels) in the pre- and post- contrast images are compared. To avoid over-estimation of the significance level due to redundant t-tests of each pixel, the FDR (false discovery rate) statistical correction is applied [11]. Each pixel in the image is assigned the significance level accordingly, resulting in a *statistical significance image* (*P*-values). Pixels with *P*-values < 0.05 are indicated as statistically significant, resulting in a *binary significance image*, where all significant pixels are assigned with 1 and the non-significant pixels with 0. The second step involved a calculation of the enhancement differences between the pre and post contrast images, which is computed for each pixel and referred to as *E* (percentage value of the intensity difference).

**Figure 1:**
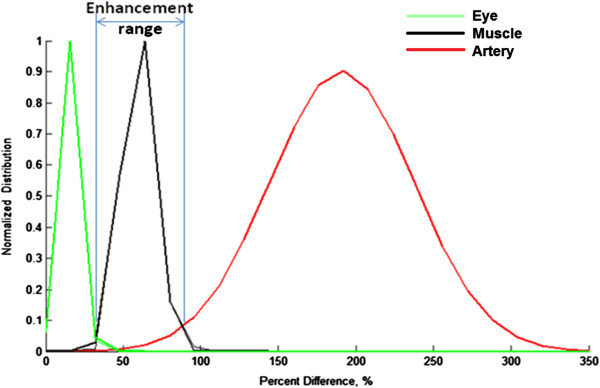
**Pre-post comparison method.** The enhancement-distribution histograms of three representing regions (ROIs): eyeball, muscle and a blood vessel. Black line represents an enhancement distribution in the muscle ROI (temporal muscle), green line for eyeball ROI (vitreous humor) and red line demonstrates blood ROI (superior sagittal sinus). Author:

The enhancement-distribution histograms of three representing regions: eyeball, muscle and a blood vessel are calculated in order to determine BBB breakdown enhancement range for each individual subject (Figure 1). Muscle is used as a normalization reference to determine intensity for a “permeable tissue” with no BBB, and the blood vessel region used to exclude pixels which represent vessels. Thus, a pixel is considered as a “permeable brain region” when its intensity significantly changed after contrast injection and it was in the range between the intact BBB (“eyeball”) and the blood vessel. In order to define an enhancement range, a Gaussian fit is applied to the histogram that makes it possible to estimate the mean *μ* and standard deviation *δ* of the enhancement distribution. Significantly different pixels (taken from the binary significant image) within the enhancement range of *μ* ±3*δ* are considered to represent brain tissue with leaky BBB (denoted as potential for leaky BBB, PBBB).

**Figure 2:**
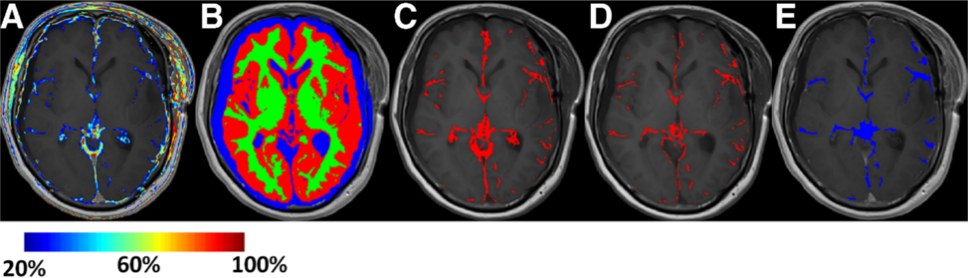
**Semi-quantitative assessment for pre-post comparison method.** (**A**) Enhancement distribution at range of 20%-100% defined with muscle ROI. (**B**) Brain masking: CSF – blue, grey matter – red, white matter – green. (**C**) Permeable pixels at region defined by total brain region mask. (**D**) Permeable pixels at region defined by brain tissue mask, excluding pixels relating to ventricles and subarachnoid space appearing in C. (**E**) Clustering procedure applied to D. Single pixels and regions smaller than minimal size of 10 pixels were removed.

The third step applies a clustering procedure on the potentially permeable pixels. Neighboring pixels are aggregated into anatomically-connected regions (Figure 2E) in order to establish a more robust assessment of the clinically-relevant brain regions with a pathological BBB. This was achieved mathematically by applying image processing operations which rendered closely-located pixels into clusters, followed by component labeling to select pixel clusters with cluster areas larger than a minimal area. For this purpose, all the objects inside the image were ranked according to their area and connectivity (4-connected). Clusters with areas smaller than the minimal one are removed from the image. As a result, regions with a high density of permeable pixels are highlighted, while single pixels are removed as potential noise artifacts.

(2) The “*linear dynamic method*”

In this approach a dynamic study is performed. There are situations that require the contrast agent to be injected before the patient enters the scanner. The time-gap between injection and the beginning of the scan can be up to several minutes (e.g., following a trans-cranial magnetic stimulation). Under such conditions, the standard two-compartment model cannot be used, since the arterial input function (AIF) cannot be estimated. Quantitative analysis can be carried out by fitting a linear curve to the dynamic scan intensities. To allow inter-scan comparisons, the time between the injection and the scan should be similar. That is, a signal s (*t*) is fitted to a linear curve: s(t) = A · t + B. The slope (A) can be interpreted as the rate of wash-in or wash-out of the contrast agent, whereas the intercept (B) represents the relative amount of contrast agent at the beginning of scanning. A “goodness of fit” map (R^2^) can also be created. Areas with different anatomical properties are hypothesized to demonstrate different parameters (i.e. blood vessels should display a relatively high intercept and a negative slope with a large absolute value; while brain with an intact BBB is expected to display a low intercept and a “close to zero” slope).

The ability to use this parametric map for both inter- and intra-subject comparison is an important goal. However it is not straightforward: firstly due to the inherent non-linearity of the MR signal intensity with respect to contrast agent concentration, which makes it impossible to compare different scans quantitatively. Secondly, variability in the administration of the contrast agent and different physiological states of the subject, affect the reproducibility of such scans.

Thus, data normalization should be considered to overcome these issues. We propose two methods which use the selection of a region of interest (ROI) as a reference. The signal in the selected ROI is then averaged and used in one of two ways:

(1) Parametric normalization: A linear curve is fitted to the averaged signal of the selected ROI, producing slope (SL_ROI_) and intercept (IN_ROI_).

(2) Temporal normalization: The temporal signal in each voxel is normalized to the corresponding signal in the ROI. A linear curve is then fitted to the normalized signal.

**Figure 3:**
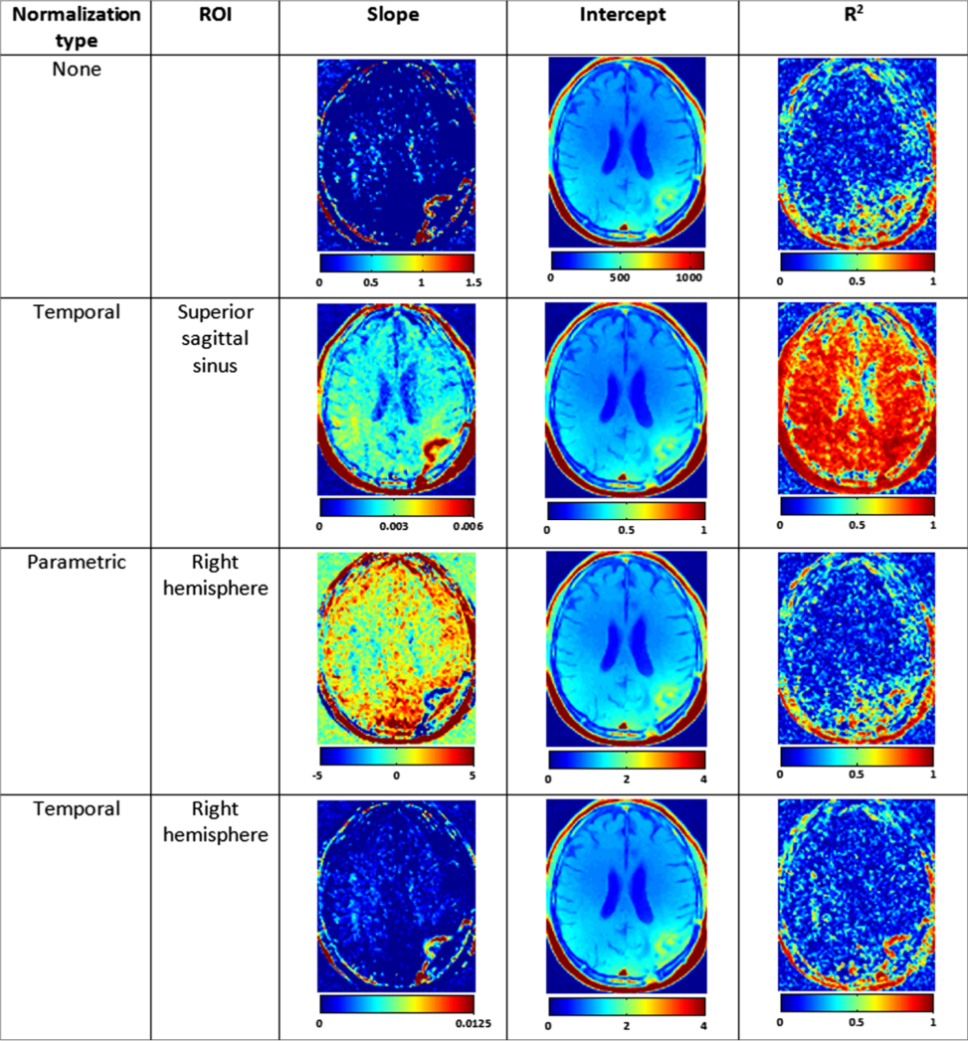
**The linear dynamic method.** A comparison of normalization methods and effect on the estimated parameters. The slope and intercept are displayed on an arbitrary scale (different scale for each method), whereas the R^2^ is always scaled in the range of 0–1. The parameters maps are smoothed using a median filter with a kernel of 3x3 voxels for display purpose.

**Figure 4:**
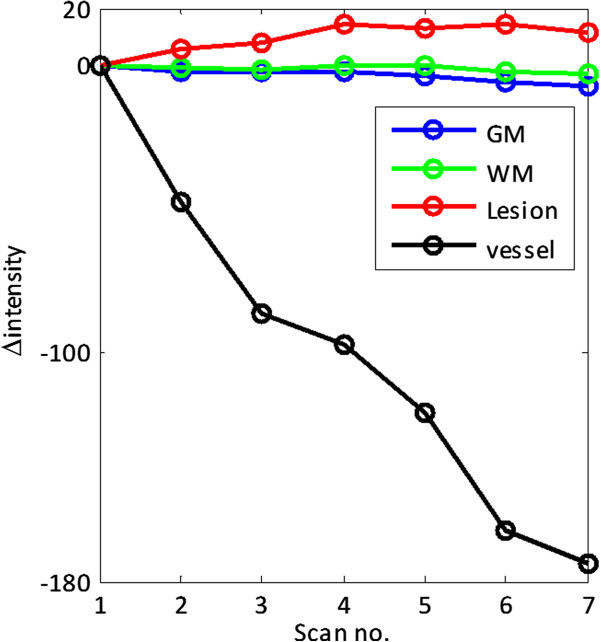
**Signal intensity changes in different tissues.** Intensity is relative to the intensity in the first scan.

Choosing the reference ROI can be done either manually or automatically. Since the need for normalization is also derived from the difference in contrast agent injection and dynamics, an option is to choose a blood vessel as the ROI. Because of its size, the superior sagittal sinus was chosen. Since the normalization is sensitive to the ROI selection, another considerable approach is to use the entire brain tissue as a reference ROI. An example of the different normalization approaches are presented in Figure 3. A patient with a glioblastoma multiforme was scanned using the following MR protocol, performed on a 1.5T Philips Intera scanner. The protocol included anatomical scan (3D gradient echo, TR/TE/TI 8.6/3.5/900 ms, FOV 240 mm, matrix 256 × 256, slice thickness 1.2 mm, 150 slices, flip angle 8°) and DCE-MRI sequence (standard spin echo, TR/TE=660/8 ms, FOV 240 mm, matrix 256 × 192, slice thickness 3 mm, 44 slices, acquisition time: 3 minutes 14 seconds). The DCE-MRI acquisition consisted of at least 7 longitudinal scans. Gd-DTPA (10 ml) followed by a saline flush, was injected approximately 3 minutes before the beginning of the scan. Since the subject had a lesion on his left hemisphere, the right hemisphere was used as ROI for the relevant calculations. As can be seen in Figure 3, the various linear fitting models provide comparable estimations. It is noticeable, however, that among the suggested methods, the temporal normalization to the blood vessel (i.e. the superior sagittal sinus) provides the better contrast and a better linear fit as indicated by the improved R^2^. Figure 4 shows the dynamic signal characteristics of various tissues. The intensity in the blood vessel, in this case the superior sagittal sinus, declined with time. Normal brain tissue remained approximately constant, while lesion area is characterized by gradual signal increase, representing contrast agent accumulation.

### Results (preliminary)

**Figure 5:**
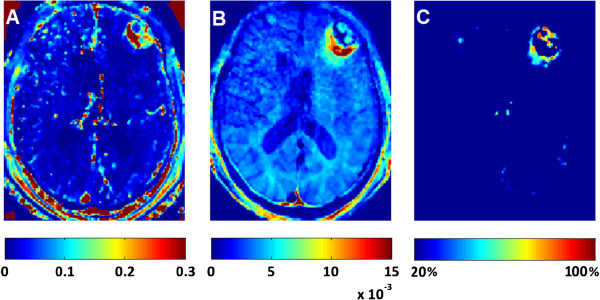
**A comparison of the permeability constant K**^**trtans**^_**,**_**( min**^**-1**^**) from Toft’s model (A), the normalized slope (sec**^**-1**^**) from the linear model (B) and the difference percentage from the post-pre method (C).** Quantitative comparison between the 3 methods is not trivial, so a qualitative comparison is shown. False color scales reflect K^trtans^ (**A**), normalized slope (**B**) and percent difference (**C**).

The two proposed methods may provide an alternative to other BBB assessment approaches, in particular for cases where DCE-MRI scanning protocol is not feasible or hard to apply. Nevertheless, these two simplified methods still call for comprehensive validation studies. Figure 5 shows a preliminary qualitative comparison between the Tofts two-compartment method (5A), the linear model (5B) and the post-pre method (5C). The three methods were applied to the same subject with a lesion on his left hemisphere, which was dynamically scanned using DCE-MRI imaging protocol. The dynamic scan was then digitally under-sampled to accommodate the data to the post-pre and linear methods. All three methods detected BBB dysfunction in and around the lesion. For the modified Tofts method, enhanced permeability is measured mostly in the core of the lesion, whereas in the post-pre and the linear fitting models the enhanced permeability appears at the margins of the lesion at even greater magnitude than in the core. This implies that while all three approaches may report “dysfunctional” BBB, differences in relation to the extent of a leaky barrier to a specific contrast agent can be revealed. The proposed clinical alternative methods may, in some circumstances, even provide more information than the accepted quantitative approach.

## Discussion and conclusion

In this manuscript we described previous and new attempts to assess BBB permeability quantitatively in humans. While the DCE-MRI has been described for more than a decade and shown to yield dysfunctional BBB under different clinical conditions, it has not been implemented in the routine clinical setup, nor tested for its power in predicting patients’ outcome, or having any contribution to clinical decision. A major limitation of DCE-MRI is the complex imaging requirements, which include initiation of the dynamic sequence prior to the intravenous injection within the magnet, followed by high frequency repeated scanning, that may force lower spatial resolution or reduced coverage of brain regions. The high temporal resolution is mainly needed for sampling the rapid passage of tracer in the arterial phase, which occurs within seconds following the injection. Although highly informative, in particular for CBF assessment, the first bolus pass of the contrast agent may be neglected while retaining sufficient information in the later stages after injection for BBB integrity assessment. A dual-temporal resolution imaging protocol has been proposed [12], based on an initial sequence with a high acquisition rate to sample the first arterial pass followed by a slower acquisition rate to sample the rest of the signal curve for a longer time. Although partially overcoming the demanding high frequency requirement, it is still based on the arterial input function and therefore tracer injection must be done during scanning. This requirement possesses complexity, which cannot always be overcome in the clinical setup, due to patient care or treatment conditions or even on-site equipment and hardware limitations. Another limitation is the low spatial resolution and the limited brain volume required when high acquisition rate is implemented. A further issue is the computation resources needed for permeability analysis of DCE-MRI, particularly if real-time analysis is desired. A full brain DCE-MRI permeability analysis can take up to an hour processing in a standard work station. The proposed new methods presented in this paper may provide a non-compromising alternative to the full dynamic methods with a much less demanding imaging protocol and computation time. In even more restricting scenarios, when the patient is unable to stay in the MR magnet for long enough periods, the post-pre semi-quantitative method may serve as an option for BBB assessment. However, it is not as yet clear whether these methods may reflect different levels of BBB dysfunction. Another issue is the lack of comparison studies between the methodologies, their sensitivity, or their ability to predict patient outcome.

## Appendix

### Quantitative analysis using DCE-MRI

In most cases, the pharmacokinetic models used for quantification of permeability from contrast-enhanced data are based on those developed for nuclear medicine. These models are based on the two-compartment exchange model (2CXM) [13] in which the contrast agent is accessible to two compartments within the overall tissue volume (“voxel”) comprising blood (“intravascular”) and tissue (“extravascular”) compartments. The latter is usually divided into the extracellular- extravascular space (EES) and the intracellular space. A common assumption is that the contrast agent is impermeable to cell boundaries so that these blood and tissue compartments essentially represent plasma and EES spaces respectively. The contrast agent is partitioned between these two compartments with concentrations of C_p_(t) and C_t_(t), respectively. The goal of contrast-enhanced techniques for permeability quantification is to measure these two dynamic quantities and thereby derive permeability-related measures according to the appropriate pharmacokinetic model. Under normal circumstances, the contrast agent is confined to the intravascular space and its passage through the volume is controlled by blood flow. However, when BBB functions are impaired, leakage will occur into the tissue (EES) compartment. This depends not only on chemical characteristics of the contrast agent, but also on the physiological characteristics of the interface between the two compartments, i.e., the BBB. Under conditions of BBB breakdown or impaired vascularity, the contrast agent can escape the vasculature and accumulate in the interstitial space of the tissue (“extravasation”) and the concentration of tracer in the tissue compartment is correspondingly increased.

As passage of the contrast agent through the blood and into the tissue reflects the competing influences of vascular perfusion and leakage, the 2CXM model enables quantification of the capillary permeability-surface area product, PS, and tissue plasma perfusion, F_p_. The “standard Tofts model” [14,15] is a simplification of this two compartment model that assumes a negligible contribution of the plasma space and therefore essentially represents a one-compartment model. The “modified Tofts model” reinstates the contribution of the plasma compartment but makes a simplifying assumption that the time taken for the contrast agent to pass through this compartment (the plasma mean transit time, MTT) is negligible [16].

Tracer-kinetic models form the quantitative basis of all compartmental models and relate the tissue and vascular concentrations of contrast agent according to the following fundamental relationship:

(1) $$C_{t}\left(t\right)=F_{p}C_{p}\left(t\right)⊗R\left(t\right)$$

where R(t) is the impulse response function (IRF) of the tissue and defines the fraction of contrast agent left in the tissue at time, t. C_p_(t) represents the “input” and is ideally measured within an arterial vessel feeding the tissue, thus representing the arterial input function (AIF). Compartmental models such as 2CXM and Tofts variants parameterize the IRF and perfusion terms in Eq. (1) in terms of fundamental physiological quantities related to the compartments and passage between them. The simplest formulation, the standard Tofts model is described by the following:

(2) $$C_{t}\left(t\right)=k_{\mathrm{trans}}C_{p}\left(t\right)⊗e^{\frac{-tk_{\mathrm{trans}}}{v_{e}}}$$

where k_trans_, known as the transfer constant, defines the rate of transfer from plasma to EES compartments in (mL/g/min), and v_e_, known as the extracellular space fraction, describes the fraction of the extravascular-extracellular space occupied by tracer in (mL/g). k_trans_ incorporates in its formulation both PS and F_p_ so that these terms cannot be dissociated. By relating to Eq. (1), it can be seen that the IRF is formulated as R_*Tofts*_(*t*) = (k_trans_/F_p_). exp(−tk_trans_/V_e_) :

The modified Tofts model adds the contribution of the plasma compartments:

(3) $$C_{t}\left(t\right)=k_{\mathrm{trans}}C_{p}\left(t\right)⊗e^{\frac{-tk_{\mathrm{trans}}}{v_{e}}}+v_{p}C_{p}\left(t\right)$$

where v_p_ is the fraction of the tissue volume occupied by blood plasma [mL/g].

Derivation of hemodynamic parameters from the concentration-time curves based on these models follow the application of principles from tracer-kinetic analysis [17]. Approaches follow either direct methods that derive some of the parameters without explicitly determining the IRF, or deconvolution methods that derive the full IRF via model-driven, model-free or parametric approaches.

The MRI approach to contrast-enhanced permeability quantification (principally DCE-MRI) calculates the concentration-time curves, C(t), of a MR-visible contrast agent from the dynamic MRI signal intensity, S(t), according to MR principles. Gd-DTPA, a freely diffusible, extracellular tracer, is the most common contrast agent in these studies whose paramagnetic Gd^3+^ core reduces the relaxation time, T1, in a concentration-dependent manner. A set of T1-weighted images is acquired, starting before a short bolus injection, and continued as uptake by the tissue followed by washout occurs. The presence of the contrast agent is indicated by an increase in signal intensity. The quantification approach relies on the assumption of a linear relationship between the T1 relaxation rate (R1 = 1/T1) and the contrast agent concentration which is believed to be valid for the relatively low concentrations expected in standard DCE-MRI studies. The most common MRI pulse sequence used in these studies is a rapid 3D gradient echo sequence for which the relationship between the signal intensity and the R1 is well-defined but non-linear. The concentration-time curves can be calculated from the dynamically acquired images if a baseline measurement of the relaxation time is performed before bolus injection. For quantitative analysis, an accurate measure of the AIF is also required. This can either be derived from a population-averaged experimental measurement [18] or directly measured in the subject within an imaging region containing a cerebral artery or within the drainage veins of the superior sagittal sinus.

## Competing interests

The authors declare that they have no competing interests.

## Authors’ contribution

YC was involved in the development of the presented methods and wrote the manuscript in collaboration with all authors. RV and GSP developed and implemented the linear dynamic method. SL developed and implemented the post-pre method. AF and IS initiated and supervised the methods development, imaging and clinical studies. All authors contributed to the writing and approved the final manuscript.
